# Engineering of Corneal Tissue through an Aligned PVA/Collagen Composite Nanofibrous Electrospun Scaffold

**DOI:** 10.3390/nano8020124

**Published:** 2018-02-24

**Authors:** Zhengjie Wu, Bin Kong, Rui Liu, Wei Sun, Shengli Mi

**Affiliations:** 1 Precision Medicine and Healthcare Research Center, Tsinghua-Berkeley Shenzhen Institute, Shenzhen 518055, China; wuzhengjiemoon@sz.tsinghua.edu.cn (Z.W.); lingxue0313@163.com (B.K.); weisun@mail.tsinghua.edu.cn (W.S.); 2 Biomanufacturing Engineering Laboratory, Graduate School at Shenzhen, Tsinghua University, Shenzhen 518055, China; liur16@mails.tsinghua.edu.cn; 3 Department of Mechanical Engineering and Mechanics, Tsinghua University, Beijing 100084, China; 4 Department of Mechanical Engineering, Drexel University, Philadelphia, PA 19104, USA; 5 Open FIESTA Center, Tsinghua University, Shenzhen 518055, China

**Keywords:** corneal tissue, electrospun, nanofibrous scaffold

## Abstract

Corneal diseases are the main reason of vision loss globally. Constructing a corneal equivalent which has a similar strength and transparency with the native cornea, seems to be a feasible way to solve the shortage of donated cornea. Electrospun collagen scaffolds are often fabricated and used as a tissue-engineered cornea, but the main drawback of poor mechanical properties make it unable to meet the requirement for surgery suture, which limits its clinical applications to a large extent. Aligned polyvinyl acetate (PVA)/collagen (PVA-COL) scaffolds were electrospun by mixing collagen and PVA to reinforce the mechanical strength of the collagen electrospun scaffold. Human keratocytes (HKs) and human corneal epithelial cells (HCECs) inoculated on aligned and random PVA-COL electrospun scaffolds adhered and proliferated well, and the aligned nanofibers induced orderly HK growth, indicating that the designed PVA-COL composite nanofibrous electrospun scaffold is suitable for application in tissue-engineered cornea.

## 1. Introduction

Cornea is the transparent part of the ocular surface; its main function is to form a barrier to protect the intraocular structure and environment, and refract light onto the retina [1,2,3]. To realize these functions, the cornea should be transparent and durable. To maintain clarity, the cornea must resist inflammation, vascularization, and invasion by surrounding cells and this requires the continuous active participation from different parts forming this deceptively simple structure. Cornea consists of five stratified layers, which include the epithelium, Bowman’s layer, stroma, Descemet’s layer and endothelium. Although these five layers contribute to the integral strength and optical properties, stroma is the most prominent layer, which is constituted by aligned collagen nanofibers. There are approximately 10 million people globally suffering from vision loss because of corneal diseases, including corneal trauma and ulceration, bacterial and viral infections and heritable conditions, every year, making it the second reason for blindness [4,5,6,7]. One primary therapy for serious corneal diseases is grafting allogenic corneal tissue, due to it has low immunological rejection. However, there is a severe shortage of high quality donor cornea which can satisfy the patients’ needs, especially with the growth of the elderly population. Other approaches have been developed due to the limitations of fresh corneal tissue, including fully artificial keratoprostheses, animal or human sources decellularized tissue, the amniotic membrane, and non-cellularized crosslinked collagen as corneal equivalents [2]. However, none of these has achieved high success and permission of tissue transplantation, even though some approaches are in current practice. Another approach is the use of fully engineered cornea with superior biocompatibility and high biological performance, such as a hydrogel system or a nanofibrous or microporous scaffold [8,9,10].

Among these approaches, the nanofibrous scaffold, fabricated by electrospinning, has evoked extensive interest and is increasingly being explored as a tissue engineered cornea because of its high porosity, surface area-to-volume ratio which can promote cells to adhere, move, proliferate and differentiate, and it can offer excellent mechanical strength, easy control of fiber properties (e.g., aligned nanofibers can simulate the lamella which is consist of aligned nanofibers in the corneal stroma), great material manipulation and implantation storability [11,12]. To date, over 200 polymers have been electrospun into nanofibers, including natural materials (e.g., collagen, gelatin, hyaluronate (HA), chitosan, silk fibroin (SF), etc.) and synthetic (e.g., polycaprolactone (PCL), poly-l-lactic acid (PLLA), poly (lactide-*co*-glycolide) (PLGA), polyethylene oxide (PEO), polyvinyl acetate (PVA), etc.) materials [13,14,15]. Among these polymers, collagen is the most commonly used material for the fabrication of corneal scaffolds, because it is the main constitute of natural corneal extracellular matrix, and it has good biodegradability, excellent biocompatibility, and low immunogenicity [16,17]. Donna et al. electrospun aligned collagen nanofibers and studied the effect of the substrate composition and aligned nanofibers on the phenotype of corneal fibroblasts. Their result indicated that cells grew on the non-aligned collagen electrospun scaffolds upregulated the expression of a smooth muscle actin protein than aligned scaffolds, and showed increased overall light scattering, suggesting that aligned collagen electrospun nanofibrous scaffolds are suitable for the replacement of corneal tissue [18,19]. However, the main drawback of the electrospun collagen scaffold is its poor mechanical properties, it does not match natural corneal tissue, and it does not meet the requirement for surgery suture, which limits its clinical applications to a large extent. Recent research results show that mechanical properties, including strength, stiffness and elasticity, are essential elements which directly affect the cell ability to adhere, proliferate and differentiate [20]. In addition, Optical transparency is a significant property that should be considered while developing a bioengineered corneal construct. A healthy cornea is required for clear vision and it contributes to two-thirds of the total refractive power of the eye, which is the most remarkable property of the cornea. For this reason, a corneal equivalent fabricated by tissue engineering should be able to transmit most of the visible light to mimic the natural behavior of the native cornea [3]. As a synthetic polymer, PVA has been widely used as a supporting material applied in corneal tissue engineering due to its transparency, flexibility and mechanical stability in contrast with conventional scaffolds fabricated by natural polymers [21,22]. Besides, PVA hydrogels are used in soft contact lenses, and PVA solution is used as an ophthalmic solution acting as artificial tears [23] due to its good biocompatibility, inherent non-toxicity, and desirable physical properties, such as its high degree of swelling in aqueous solutions and its rubbery or elastic nature [24].

In this study, to fabricate a tissue engineered corneal scaffold with good mechanical properties and biocompatibility, the blend of collagen and PVA solution were electrospun. Aligned and non-aligned blend scaffolds with two different blend ratios are fabricated. The effect of the PVA addition amount and the degree of alignment on the mechanical properties and light transmittance of the PVA-COL composite electrospun scaffolds are studied. Human keratocytes (HKs) and human corneal epithelial cells (HCECs) are inoculated on these scaffolds and cultured for four weeks, and the effects of the materials and topological structure on these cells are studied.

## 2. Materials and Methods

### 2.1. Materials

Type I collagen (molecular weight: 280,000 g/mol) from porcine skin was purchased from Sichuan Ming rang Co. (Chengdu, China). PVA (molecular weight: 80,000 g/mol), 1,1,1,2,2,2-hexafluoro-2-propanol (HFIP), glycine, glutaraldehyde, phosphoric acid and acetic acid were obtained from Sigma-Aldrich (Irvine, UK).

### 2.2. Electrospinning

The collagen solution (7% *w*/*v*) was prepared by dissolving it in HFIP under magnetic stirring for 48 h at room temperature. The PVA solution at 10 and 15 wt % was achieved by dissolving in 80 °C distilled water for 4 h and stirring until complete homogeneity. Collagen solution at 6.3 mg/mL and 25 mg/mL were prepared by dissolving it in 1 M acetic acid. A 7% PVA-collagen (PVA-COL) blend solution was prepared by mixing 15% PVA and 6.3 mg/mL collagen, with a volume ratio of 45:55, and a 9% PVA-COL blend solution was prepared by mixing 15% PVA and 25 mg/mL collagen with a volume ratio of 13:12. The optimal spinning conditions for the 7% collagen, 7% PVA-COL, and 9% PVA-COL aligned nanofibers were as follows: needle collector distance, 150, 170, and 170 cm; voltage applied to a blunt, 12, 13, and 15 kV; flow rate, 0.3, 0.3, and 0.2 mL·$h^{-1}$; rotating speed, 300 rpm. Use 23 gauge needle tip of a 5 mL syringe, and 3 h of spinning time. The humidity was regulated to 50%, and temperature to 25 °C. The electrospun setup used to fabricate the aligned nanofibers has been described in detail by our previously published paper [25]. The non-aligned nanofibers were obtained by using a low speed cylinder collector. The newly fabricated electrospun mats were separated from the collector and vacuum dried at 50 °C for 24 h.

### 2.3. Crosslinking the Electrospun Fibers for Cell Culture

All electrospun mats were crosslinked by a phosphoric acid vapor for one day and glutaraldehyde vapor for two days as described previously [19,25], to improve the water resistance ability. After that, electrospun mats were soaked in 0.1 M glycine overnight to remove the excess glutaraldehyde. Samples were then transferred to culture dishes and rinsed with de-ionized water and sterilized under UV light.

### 2.4. SEM Observation and Mechanical Properties

The electrospun scaffolds were dried by supercritical carbon dioxide extraction (5 × 5 min, 1 × 20 min). Samples were coated with a 20 nm-thick layer of platinum and examined by SEM (SEM, S-4800 FESEM, HITACHI, Japan) at 3 kV. The images were digitized and analyzed by Image Pro Plus (IPP) software to determine the average fiber diameters and degree of alignment. Six samples were measured for each group of electrospun mats. Sixty measurements of each sample were calculated for the average value and standard deviation. All measurements were calibrated using the scale on the micrograph as a reference for diminution of errors in calculating the magnification of the scanned photos.

Mechanical tests of aligned 9% PVA-COL, 7% PVA-COL, 7% collagen and non-aligned 9% PVA-COL were determined using a mechanical testing machine (Electropuls E3000, Instron, Norwood, MA, USA). Samples were prepared by immersing the samples into PBS for one day at 37 °C and then cutting them in a rectangular shape (30 mm × 5 mm). Three specimens were tested in different solutions at aligned or non-aligned orientations. Each sample was repetitively loaded and unloaded three times at the same load level. It was found that the elongation ratio of the unloading curve is larger than that of the loading curve. When the load is unloaded to zero, the testing mat can be restored to its original shape. After this preconditioned, the load–deformation curve of each sample was detected.

Loading cycles were performed using a load sensor of 3000 N and a loading speed of 2 mm∙$\min^{-1}$. The stress–strain curve of the electrospun scaffold was constructed from the load–deformation curve.

### 2.5. Light Transmittance

The aligned 9% PVA-COL, 7% PVA-COL, 7% collagen, 10% PVA and non-aligned 9% PVA-COL electrospun scaffolds were cut into 6.5 mm circles by using a trephine to fit the well scale in a 96-well plate. These samples were then immersed into PBS for one day placing in 96-well plates; the blank control was PBS. A microplate reader (Multiskan Spectrum, purchased from Thermo Scientific, Waltham, MA, USA) was then used to measure the constructs light transmittance under the wavelengths between 400 nm and 650 nm. The transmittance values were then calculated by the equation as mentioned previously [26]: $T\left(\%\right)=10^{-A}\times 100$, where A and T are absorbance and transmittance, respectively.

### 2.6. Cell Culture

HCECs [27,28,29,30,31] were purchased from the RIKEN BioResource Center (Tsukuba, Japan), and HKs [32,33] were obtained from the He Eye Hospital (Shenyang, China). These two cell types were cultured in Dulbecco’s modified Eagle’s medium (DMEM)-F12 (50:50, *v*/*v*, Invitrogen, Carlsbad, CA, USA) under 5% CO_2_ at 37 °C. The cell medium was supplemented with 10% fetal bovine serum (FBS; HyClone, Logan, UT, USA), 100 U/mL penicillin and 100 μg/mL streptomycin (Invitrogen) and recombinant human epidermal gro wth factor (10 ng/mL, Thermo Fisher, Waltham, MA, USA); the medium for the HCECs also contained bovine insulin (5 μg/mL, Thermo fisher). The cells were subcultured using trypsin (0.25%, Invitrogen) upon reaching approximately 80% confluence. The culture media was changed every 2–3 days.

### 2.7. Cell-Seeded Electrospun Scaffolds

The 9% PVA-COL (aligned and non-aligned) and the 7% aligned collagen electrospun mat were sterilized by exposing them to UV light for 1 h after immersing them in 75% alcohol for 2 h. Then, these scaffolds were coated with 0.01% poly-l-lysine overnight to improve the cell adhesion before transferring them to the 96-well plates. One hundred microliters of HCECs suspension (2 $\times 10^{5}$ cells) and HKs suspension (2 $\times 10^{5}$ cells) were added into these scaffolds respectively and each added 100 μL fresh culture medium tomorrow. The culture media were changed every 2 days.

### 2.8. Cell Proliferation Assay

The proliferation of HKs and HCECs on the electrospun scaffolds were measured using Cell Counting Kit-8 (CCK-8, DOJINDO) based on the manufacturer’s guidance. Briefly, the scaffolds in Section 2.6 were washed in PBS for three times. Then, 500 μL of culture medium and 50 μL of CCK-8 were added to each culture dish and were incubated in the dark for 2 h at 37 °C. After incubation, 500 μL culture medium was transferred to a 96-well plate, and the optical density (OD) at 450 nm was measured immediately. The cells seeded in the culture dish as a control group were treated the same way, and the CCK-8 medium was used as the blank control. Three samples were tested for each group.

### 2.9. Observing the Fluorescently Labeled Cells on the Scaffolds

The red fluorescent protein (RFP) and the green fluorescent protein (GFP) labelling kits were purchased from Shanghai Zhongqiao Xinzhou Co. (Shanghai, China). According to the manufacturer’s instructions, the fluorescently labeled cells continuously express fluorescent protein after proliferation.

Fluorescently labeled HKs (red) and HCECs (green) were used to observe the proliferation of these cells on the electrospun scaffolds and the effect of the aligned and non-aligned topological structure on cells was observed by confocal microscopy (Xcellence, Olympus, Hong Kong, China). The method for the cell seeding is described in Section 2.6. After 1, 2, 3, and 4 weeks of culture, confocal microscopy was used to observe the HKs and HCECs on the hybrid scaffolds by adjusting the focal plane and excitation wavelength.

### 2.10. Statistical Analysis

All results are shown as the mean ± standard deviation (SD). The statistical analyses were executed by using one-way ANOVA and two-way ANOVA with a Bonferroni post-hoc test to measure the significance degree, where * *p* < 0.05 was considered statistically significant.

## 3. Results

### 3.1. Morphology of the Electrospun Scaffolds

**Figure 1:**
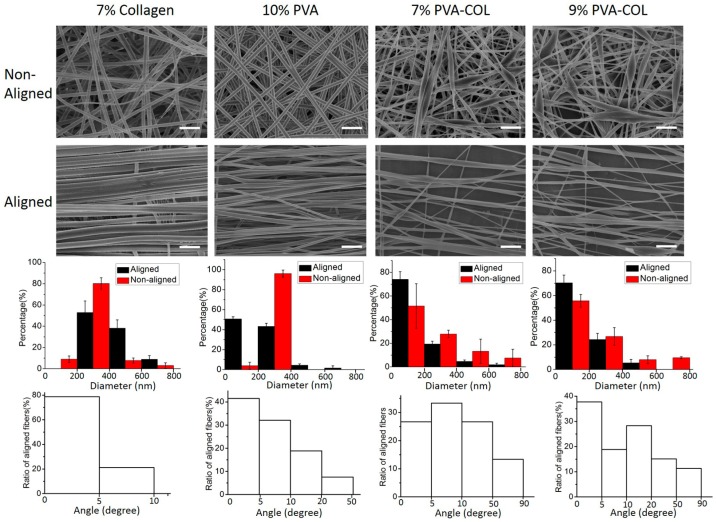
SEM images of the non-aligned and aligned electrospun nanofibers, the diameter range and the alignment degree histograms of the respective electrospun scaffolds. Scale bar, 2 μm.

The designed parallel double thin plates collectors with an independent fiber removal device were used to obtain aligned fibers, which has been described in detail by our previously published paper [25]. The SEM images of the 7% collagen (aligned and random), 10% PVA (aligned and random), 7% PVA-COL (aligned and random) and 9% PVA-COL (aligned and random), the diameter range histogram of the above aligned and random fibers, and the alignment degree histogram of the above aligned fibers which were shown as the average value calculated from six samples, are shown in Figure 1, and their average diameter is shown in Table 1. From the SEM images and the histogram of the diameter range, we found that the fibers of pure collagen and pure PVA were more uniform than the PVA-COL composite nanofibers, and this may because the hybrid of collagen and PVA makes the mixture not as homogeneous as the pure one. The average fiber diameter of the random 7% PVA-COL nanofibers was 211.6 ± 142.5 nm, the diameters of 51.6 ± 18.8% of the random nanofibers were between 0 and 200 nm, 27.8 ± 3.2% between 200 and 400 nm, 13.3 ± 10.3% between 400 and 600 nm, and 7.5 ± 7.3% between 600 and 800 nm. The diameter of the aligned 7% PVA-COL was a little smaller than that of the random ones (183.3 ± 96.7 nm) and the diameter distribution of the aligned nanofibers was also narrower than that of the random ones: 74 ± 6.5% were between 0 and 200 nm and the rest were between 200 and 800 nm. The average fiber diameter of the random 9% PVA-COL nanofibers was 262.9 ± 199.3 nm, and the diameters of 55.7 ± 5.1% of the random nanofibers were between 0 and 200 nm, 26.8 ± 7.1% between 200 and 400 nm, 7.9 ± 3.2% between 400 and 600 nm and 9.5 ± 0.8% between 600 and 800 nm. The diameter of the aligned 9% PVA-COL was smaller than that of the random ones (163.1 ± 103.2 nm) and the diameter distribution of the aligned nanofibers was also narrower than that of the random ones: 70.2 ± 6.4% were between 0 and 200 nm and the rest were between 200 and 600 nm. Thus, the aligned PVA-COL nanofibers were smaller and more homogeneous than the random ones, which was in accordance with previous research results [34,35]. From the SEM images and the alignment degree histogram, we found that the alignment degrees of pure collagen and PVA nanofibers was higher than that of the composite nanofibers, and this may result from the increased charge improving the instability of whipping, resulting in the non-uniform splitting of the jet [36].

### 3.2. Mechanical Properties and the Light Transmittance of the Electrospun Scaffolds

**Figure 2:**
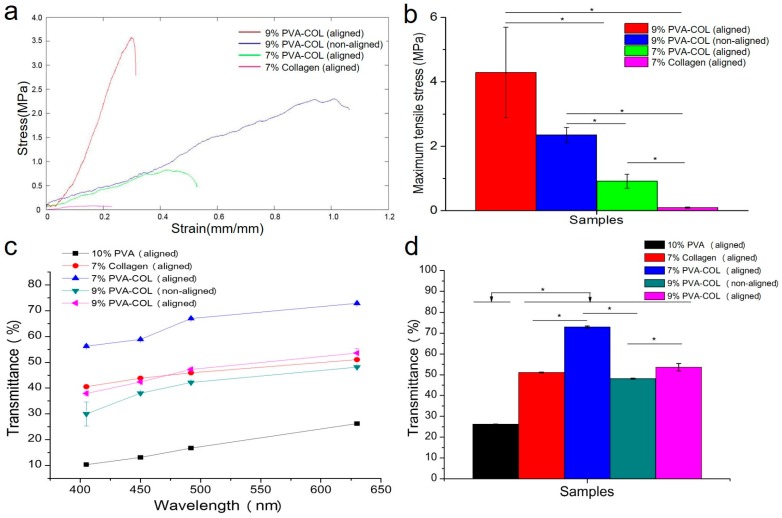
Mechanical properties and transparency test results: (**a**) average strain–stress curves of the 9% PVA-COL (aligned and non-aligned), 7% aligned PVA-COL and collagen electrospun scaffolds; (**b**) maximum tensile stress histogram of the samples; (**c**) the average transmittance of the 10% aligned PVA, 7% aligned collagen, 7% aligned PVA-COL and 9% PVA-COL (aligned and non-aligned) electrospun scaffolds under the wavelengths of 405, 450, 492 and 630 nm; and (**d**) transmittance histogram of the samples under the wavelength of 630 nm. The data represent the means ± SD (* *p* < 0.05).

The mechanical properties of the electrospun blend nanofibrous mats are strongly influenced by the properties of each polymer in the blend nanofibrous mats, nanofiber structure, and the interaction between each polymer nanofiber [37]. The tensile strain–stress curves of the crosslinked 9% PVA-COL (aligned and random), 7% aligned PVA-COL and 7% aligned collagen electrospun mats are shown in Figure 2a,b. The maximum tensile strength of pure collagen is just 0.112 MPa, which is in accordance with the study result by Torbet [38], indicating that the pure collagen electrospun mat is not suitable for the application as a tissue engineered cornea. After mixing with PVA, the mechanical properties of the hybrid scaffold improved. The maximum tensile strength of the 7% aligned PVA-COL was 0.863 MPa. For the 9% PVA-COL scaffolds, the non-aligned nanofiber tensile strength was 2.252 MPa, and the aligned nanofibers significantly improved to 3.581 MPa, which was similar to that of the natural corneal tissue at approximately 3–5 MPa, and the strain at break was approximately 0.192 [39,40]. Differences in the tensile properties between the random and the aligned nanofibers are seen in other materials such as pure collagen, PCL and PLGA [35,41]. The mechanical property of 7% PVA-COL electrospun mat (0.863 MPa) still cannot match natural corneal tissue and does not meet the requirement of tissue-engineered cornea scaffold. Our intention is adding PVA to increase the mechanical property of the scaffold. Therefore, 7% PVA-COL electrospun mat is not selected to do the cell experiment.

Figure 2c shows the light transmittance of the 10% aligned PVA, 7% aligned collagen, 7% aligned PVA-COL and 9% PVA-COL (aligned and random) electrospun scaffolds under the wavelengths of 405, 450, 492 and 630 nm. The light transmittance of pure PVA is very poor, with the highest at 26.222 ± 0.114% under 630 nm wavelength. The pure collagen electrospun scaffold had the highest light transmittance, 51.012 ± 0.221% at 630 nm wavelength, which was higher than that of the pure PVA mat. For the PVA-COL electrospun mat, the highest light transmittance of the 9% PVA-COL at 53.570 ± 1.843% was lower than that of the 7% PVA-COL electrospun scaffold at 72.891 ± 0.475%, indicating that the light transmittance was related to the amount of PVA, and the higher amount of PVA, the lower the light transmittance of the composite scaffold. The alignment degree of the fibers also affected the light transmittance. In Figure 2d, the 9% aligned PVA-COL scaffold showed a light transmittance of 53.570 ± 1.843% at 630 nm wavelength, while the light transmittance of the random scaffold was just 48.121 ± 0.195% in the same condition, revealing that the light transmittance of the nanofibrous scaffolds increased with the increased alignment degree [42].

### 3.3. Proliferation of Cells on the Electrospun Scaffolds

**Figure 3:**
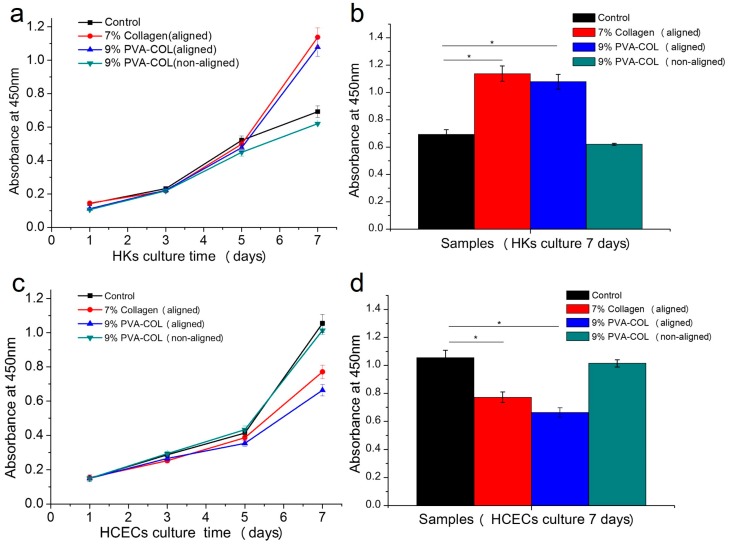
The proliferation results of the HKs and HCECs: (**a**) proliferation of HKs on the 7% aligned collagen and the 9% PVA-COL (aligned and non-aligned) electrospun scaffolds within seven days; (**b**) proliferation histogram of the HKs in the different electrospun scaffolds cultured for seven days; (**c**) proliferation of HCECs on the 7% aligned collagen and the 9% PVA-COL (aligned and non-aligned) electrospun scaffolds within seven days; and (**d**) proliferation histogram of the HCECs in the different electrospun scaffolds cultured for seven days. The control group was the cells that were seeded on the culture plate, the data represent the means ± SD (* *p* < 0.05).

The HKs and HCECs were inoculated in the 7% aligned collagen and the 9% PVA-COL (aligned and random) electrospun scaffolds, respectively, and were cultured in 96-well plates. CCK-8 was used to examine cell proliferation after culturing for 1, 3, 5 and 7 days. The control group was the cells seeded on the culture plate. Figure 3 shows the proliferation of the HKs and HCECs qualitatively determined by the optical density (OD) value at 450 nm. The OD values showed an apparent increase for all scaffolds with the increase of culture time, indicating that the electrospun scaffolds had good biocompatibility, with no toxicity to the cells.

As shown in Figure 3a,b, the proliferation activity of the HKs seeded in electrospun mat showed no apparent difference compared to the control group cells after culturing for five days, suggesting that cells can adhere and proliferate in all three scaffolds. For the control group, the cells were not subjected to the effect of contact inhibition because the cells did not spread over the culture well. After culturing for seven days, the cells in the control group already covered the whole well, and the proliferation activity decreased due to contact inhibition. However, the cells seeded in the electrospun mat maintained high proliferation activity because the electrospun scaffolds had higher surface area-to-volume ratio, with a wider space for the cells to adhere and proliferate. The alignment degree of the nanofibers also had a prominent influence on the proliferation activity of the HKs. Compared to the aligned collagen and PVA-COL scaffold, the HKs seeded on the random PVA-COL scaffold had a lower proliferation activity, with an OD value range that was far less than that of the aligned scaffolds, indicating that the aligned nanofibers were more suitable for the long-term culture of HKs. In Figure 3c,d, the proliferation activity of the HCECs seeded in the electrospun mat showed no apparent difference compared with the control group cells when they were cultured for five days, suggesting that the cells adhered and proliferated in all three scaffolds. After culturing for 5–7 days, the cells seeded on the PVA-COL random scaffold proliferated more actively than those of the other aligned scaffolds.

### 3.4. Fluorescently Labeled HKs on the Electrospun Scaffolds

It is common knowledge that cells react differently to micro-topography and nano-topography, and can be guided by using physical cues to mimic natural tissues [43,44]. It is suggested that some cells distribute randomly within a non-woven scaffold and grow orderly on an aligned scaffold. The fluorescently labeled HKs and HCECs were inoculated on the aligned and non-aligned collagen and the PVA-COL electrospun scaffolds to determine the biocompatibility of the composite materials and the effect of the fiber alignment degree on the behavior of the HKs and HCECs.

**Figure 4:**
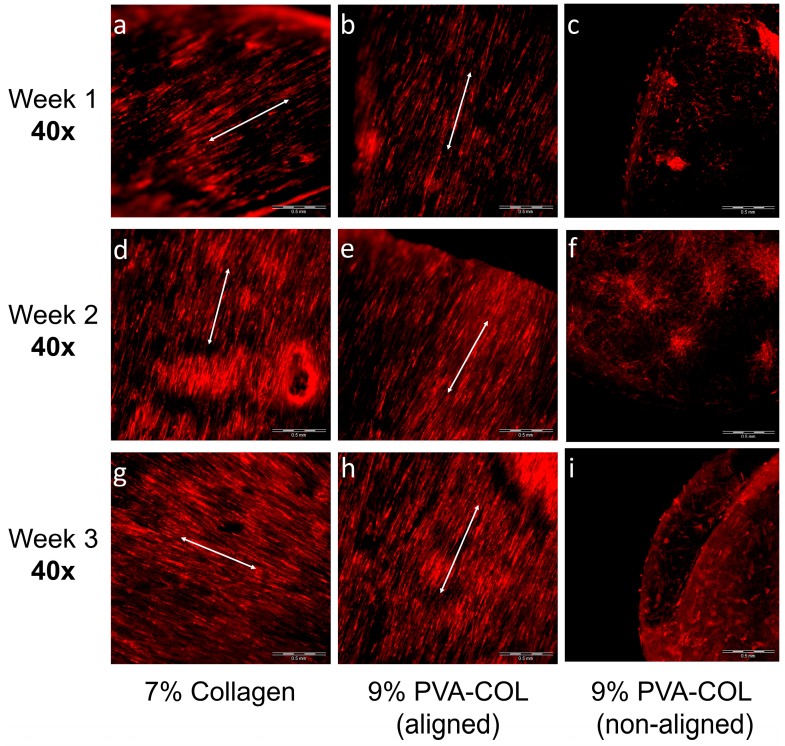
RFP labeled HKs: on the 7% aligned collagen (**a**,**d**,**g**); and the 9% PVA-COL aligned (**b**,**e**,**h**); and non-aligned (**c**,**f**,**i**) scaffolds that were cultured for: one week (**a**–**c**); two weeks (**d**–**f**); and three weeks (**g**–**i**). The scale bar is 500 μm.

The RFP labeled HKs seeded on the 7% aligned collagen and the 9% PVA-COL (aligned and non-aligned) scaffolds were cultured for one, two and three weeks and are shown in Figure 4: Figure 4a,d,g shows cells cultured on a 7% aligned collagen electrospun scaffold for one, two and three weeks, respectively; Figure 4b,e,h shows cells on a 9% aligned PVA-COL electrospun scaffold; and Figure 4c,f,i shows cells on a 9% random PVA-COL electrospun scaffold. The cell numbers increased on all three scaffolds, as shown in Figure 4. In Figure 4a–c, when culturing for one week, the HKs cells adhered and proliferated on the three scaffolds, and the cells on the aligned collagen and PVA-COL scaffolds distributed orderly along the orientation of the fibers. However, the cells on the random PVA-COL scaffold distributed randomly, with no orientation. In Figure 4d–f, when cultured for two weeks, the cells on the aligned scaffolds maintained a directional alignment and the cells remained randomly distributed on the random scaffold, and the rehydration of the electrospun scaffolds decreased the clarity of the random scaffold. When culturing for three weeks, the result was the same as for two weeks. From these results, it was concluded that both the 7% collagen and 9% PVA-COL electrospun scaffolds had good biocompatibility and supported the cells to adhere and proliferate. The arrangement (aligned or random) of the electrospun scaffolds did not affect the adhesion of the cells, but largely influenced the cell growth pattern. The aligned nanofibers induced the aligned growth of the HKs, and the random fibers did not induce the aligned growth. These results are in accordance with those by Wu [6] and Zhang [45]. Both the 9% aligned PVA-COL and 7% aligned collagen scaffold induced orderly HKs growth, and there was no apparent difference between the cell proliferation activity and the growth pattern of the cells on both scaffolds.

**Figure 5:**
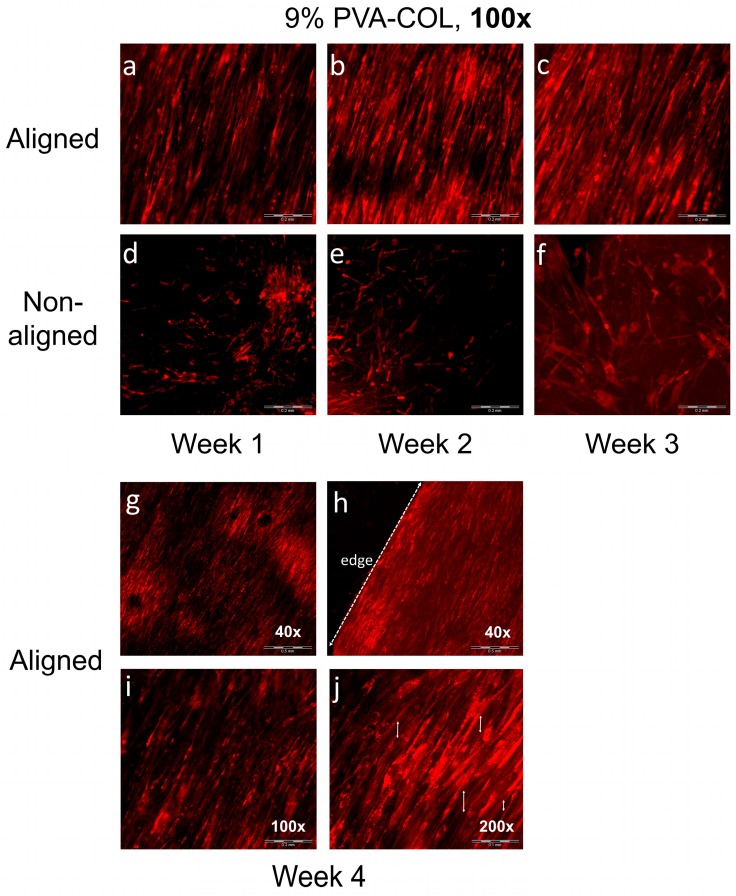
The HKs growth on the 9% PVA-COL scaffolds: (**a**–**c**) on the aligned scaffolds that were cultured for one, two and three weeks; and (**d**–**f**) on the random scaffolds that were cultured for one, two and three weeks. The scale bar is 200 μm. (**g**–**j**) The aligned 9% PVA-COL scaffolds after culturing for four weeks. Scale bar: (**g**,**h**) 500 μm; (**i**) 200 μm; and (**j**) 100 μm.

To further study the effect of fiber arrangement on the cells, we determined the cell growth pattern on the 9% aligned and random PVA-COL scaffolds under high magnification (100×) after culturing for one, two and three weeks, as shown in Figure 5a–f. The cells seeded on the aligned nanofibers distributed orderly along the orientation of the fibers, and distributed randomly on the non-aligned scaffold. The radial length of the cells on the aligned scaffold was 3.5 ± 0.2-fold of that on the random scaffold, with a spindle-like shape for the cells on the random scaffold. In addition, the cells distribution on the aligned scaffold was more homogeneous, with the cells nearly covering the whole surface of the scaffold.

Finally, we tracked the HK growth on the 9% aligned PVA-COL scaffold after culturing for four weeks and observed the cell morphology and growth pattern under magnifications of 40×, 100× and 200×, as shown in Figure 5g–j. Under the 40× magnification shown in Figure 5g,h, most cells adhered and proliferated on the scaffold, completely merging and arranging orderly along one orientation. Figure 5h also shows the edge of the scaffold and petri dish, indicating that the cells only adhered and proliferated in the scaffold. In the higher magnification images shown in Figure 5i,j (100× and 200×, respectively), it was clearly observed that the cells were joined with periphery cells, merging into entire multi-cell sheets, and the cells between the different layers also had junctions.

### 3.5. Fluorescently Labeled HCECs on the Electrospun Scaffolds

**Figure 6:**
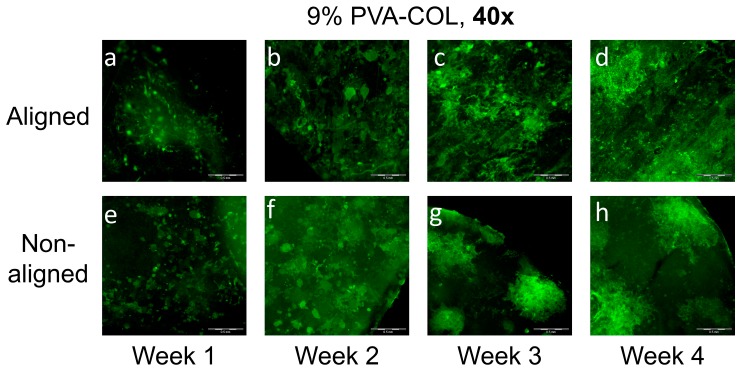
The GFP labeled HCECs on the: aligned (**a**–**d**); and non-aligned (**e**–**h**) 9% PVA-COL scaffolds after culturing for: one week (**a**,**e**); two weeks (**b**,**f**); three weeks (**c**,**g**); and four weeks (**d**,**h**). The scale bar is 500 μm.

The green florescence protein (GFP) labeled HCECs seeded on the 9% PVA-COL are shown in Figure 6: Figure 6a–d shows cells cultured on the aligned scaffold for one, two, three and four weeks, respectively; and Figure 6e–h shows cells on the random scaffold for one, two, three and four weeks, respectively. After culturing for one week, the cells on the random scaffold had a better adhesion and higher proliferation rate than those on the aligned scaffold, and most cells on the random scaffold were large and had a flat shape, while on the aligned scaffold, most cells tended to be round. This was in accordance with the result that the cells on the random scaffold had a higher proliferation activity when they were cultured for five days compared to those cultured on the aligned scaffold as determined by CCK-8. However, when culturing for two weeks, the cells on the aligned scaffold proliferated rapidly and spread on the fibers. Additionally, the local cells already formed a cell sheet. After culturing for three and four weeks, the cells already nearly covered the surface of the aligned scaffold, not only along the X and Y dimensions, but also had the cell stack in the Z direction, while the cells distributed non-uniformly on the random scaffold. Moreover, compared with the HKs, the cells on the aligned scaffold did not arrange orderly, indicating that the aligned nanofibers did not induce the aligned growth of HCECs, which is in accordance with that by Yan et al. [40].

**Figure 7:**
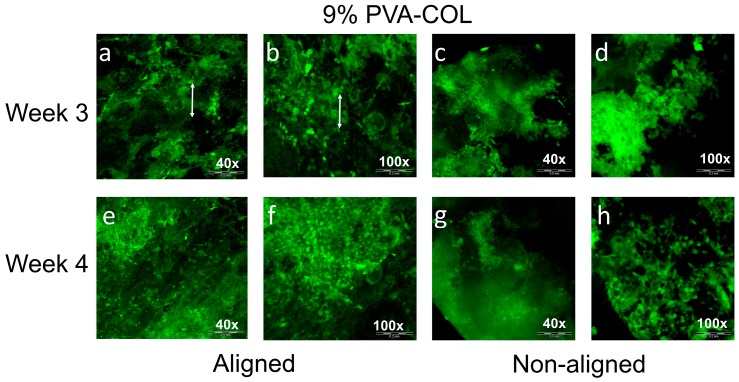
The HCECs on the: aligned (**a**,**b**,**e**,**f**) and non-aligned (**c**,**d**,**g**,**h**) 9% PVA-COL scaffolds after culturing for: three weeks (**a**–**d**); and four weeks (**e**–**h**). Scale bar: (**a**,**c**,**e**,**g**) 500 μm; and (**b**,**d**,**f**,**h**) 200 μm.

Similar to the HKs, we also observed HCECs growth on the aligned and non-aligned (Figure 7) 9% PVA-COL scaffolds under the different magnification when the cells were cultured for three and four weeks. As shown in Figure 7a,b (40× and 100×, respectively), when they were cultured on the aligned scaffolds for three weeks, the cells were large and covered the whole scaffold. The cell junctions formed a small cell sheet, and a large sheet further. The local sheet expanded along the Z direction and formed multi-layers. As shown in the 100× image, the cells grew randomly and the aligned nanofibers did not induce the aligned growth of the HCECs. When culturing for four weeks, as shown in Figure 7e,f (40× and 100×, respectively), the cells cover rate showed no apparent difference compared with three weeks. However, the local cells in Figure 7f were small, had a round shape and a high density, and this might because of the contact inhibition result from the lack of the expansion space for the cells, indicating that cell proliferation was inhibited when culturing on the aligned nanofibers for over four weeks. When cultured on the non-aligned scaffolds, as shown in Figure 7c,d at three weeks and Figure 7g,h at four weeks, the HCECs covered most of the region of the scaffold, but they were not evenly distributed and tended to aggregate in the local area.

## 4. Discussion

Human corneal stroma is the most prominent layer of the cornea, which processes a unique organization of collagen fibrils with a mono-dispersed fibril diameter and uniform local interfibrillar spacing [1,2,3,4,5,6,7]. The stroma contains over 200 acellular collagenous lamellae. The nano-scale collagen fibers are arranged parallel to each other, perpendicular to those of the adjacent lamellae [1,2,3,4,5,6,7]. As for engineering corneal tissue, the chemical composition of substrate materials is a cue that could affect extracellular matrix (ECM) elaboration. Several studies have noted the significance of selecting appropriate proper scaffold material of tissue-engineered cornea applications, as materials can make a difference in stromal bioengineering, and many synthetic polymers have been shown to be cytotoxic and induce inflammatory responses from the cells [46,47]. Gill et al. and Lawrence et al. reported that corneal fibroblasts can be induced by the surface-patterned silk films to elongate [48,49,50]. Other studies, using chemically crosslinked collagen, magnetically aligned collagen, and compressed collagen gels to construct tissue-engineered cornea scaffolds, all showed that collagen is a good natural material for corneal application [51,52,53]. To mimic the highly ordered fibrous structure, electrospinning was chosen to produce fibrous structure with controlled fiber diameter and orientation [9,10]. However, pure collagen electrospun mat has poor mechanical properties and the content of collagen would dramatically influence its fiber diameter [6,15,18,19]. Wray et al. noted that the average diameter of the collagen fibers increased with the solution concentration [19]. The 7.5% collagen solution fabricated an average fiber diameter of 440 ± 70 nm [19], which was similar to our result, as shown in Figure 1 (7% collagen aligned). Thus, they suggested using a smaller collagen concentration to fabricate smaller diameter collagen fibers. To improve the mechanical properties of collagen electrospun scaffold and increase its content in the scaffold without extremely enlarging its fiber diameter, PVA was chosen to produce fibrous scaffolds with collagen. PVA is transparent and has good mechanical properties which is suitable for keratoprostheses [23]. Miyashita et al. used PVA to produce hydrogel, and immobilized collagen on it to improve its low cell affinity. They found that HCECs grew well in the PVA-COL gel, suggesting that it is possible to use PVA as a biocompatible material for engineering corneal tissue [54].

In this study, a novel electrospinning setup we have designed before was used [25], aligned and non-aligned composite PVA-COL electrospun scaffolds were fabricated. The proliferation and behavior of cells that were cultured on PVA-COL substrate are similar to that on pure collagen substrate, indicating that PVA-COL composition is suitable for culturing HKs or HCECs. Comparing with pure collagen and PVA-COL scaffolds, the maximum tensile strength was dramatically increased from 0.112 MPa to 3.581 MPa, which is similar to that of the natural corneal tissue. Moreover, the diameter of the aligned 9% PVA-COL fibers (163.1 ± 103.2 nm) was smaller than 7% pure collagen aligned fibers, even though 9% PVA-COL scaffold contains higher collagen solution concentration. Results demonstrate that using PVA-COL mix solution for electrospinning may increase the content of collagen without dramatically influence to fibrous diameter and improve pure collagen’s poor mechanical properties [18,19].

Different proliferation and morphology results of HKs and HCECs that were cultured on aligned and non-aligned electrospun scaffolds, also demonstrate that scaffold structure and fibrous alignment may affect cell behavior. As shown in Figure 4 and Figure 5, aligned or non-aligned PVA-COL fibrous mats support the cellular growth of HKs, but only aligned fibers caused HK to elongate along the axis of the fiber alignment. HKs on non-aligned fibrous scaffold did not have a preferred direction of alignment, suggesting that the HK did respond to substrates with a microstructure and organization, cells align themselves along the direction of fiber alignment. The growth of the HCECs was not controlled by the nanostructure of the cell cultured substrate. The HCECs grew in a random direction even those cultured on the aligned fibrous electrospun mat, as shown in Figure 6 and Figure 7. Teixeria et al. noted that compared with epithelial cells, substrate topography has a more effective influence to keratocytes, after aligning corneal keratocytes in one direction by using silicon wafers with micro- and nanosize grooves [55]. Vrana also reported that, for scaffolds upon which keratocytes are cultured, patterned substrates have better transparency than those scaffolds with unpatterned substrate [56]. This might relate to the natural truth that, in vivo, keratocytes are grown in layers of aligned collagen fibers in the stroma, suggesting that, by mimicking the natural high aligned microenvironment of stroma, it is possible to fabricate tissue-engineered cornea in vitro.

How does the structure of substrate influence the cultured cell behavior? Teixeria et al. etched parallel ridges in silicon oxide-coated wafers, and cultured HKs on them to test the effect of substrate micro-structure on cell behavior [55]. They found that the larger is the ridge separations, the more orderly aligned the keratocytes grow, and suggested that substrates with 800 nm or larger ridge separations are better to induce cell behavior than the one with 400 nm. Lindsay et al. and Donna et al. seeded corneal fibroblasts on collagen electrospun scaffolds, and found that substrates with smaller fiber diameter (e.g., 40 nm) support cells aligning in the direction of the fiber orientation [18,19]. Jian et al. reported that a substrate of aligned polyester urethane urea fibers with 165 ± 55 nm diameter, induced cultured human corneal stromal stem cells aligning [6]. According to previous studies, it seems that the fibrous diameter of the scaffold would influence the cultured cell behavior, and whether the fibers with smaller or bigger diameter is good for inducing cell aligning, still has debate. In our study, the results show that there was no apparent difference between the cell proliferation activity and the growth pattern of the cells on both 7% collagen substrate with 431.8 ± 72.6 nm fiber diameter and 9% PVA-COL one with 163.1 ± 103.2 nm fiber diameter. The differences among these studies indicate that cells on the substrate may respond to scaffold’s microstructure and organization, no matter which fabricating method was used. Fiber diameter of the electrospun mat has little influence on cell behavior. HK can align strongly on the aligned fibrous electrospun mats with different fiber diameters.

Since PVA-COL can improve the mechanical properties of electrospun scaffold, and its highly ordered structure can induce the ordered growth of HKs, it suggests the possibility of using PVA-COL aligned electrospun scaffold to further mimic corneal stroma’s elegant ultrastructural organization. Thus, the next option for engineering corneal tissue might further mimic multiple interlaced lamellae of natural cornea tissue and replicate the natural arrangement of fibers in the cornea [1,2,3,4,5,6,7]. It requires building a tailored three-dimensional electrospun scaffold with different layers, stacked on each other at various angles ranging from 0° to 90°, whose physical mechanical properties and optical transparency would be more similar to the natural corneal tissue.

## 5. Conclusions

In this study, aligned composite PVA-COL electrospun scaffolds were successfully fabricated and used as a tissue-engineered corneal substitute. By adding PVA, the mechanical properties of the PVA-COL electrospun scaffolds were improved enormously, and the 9% PVA-COL aligned nanofibers showed a similar mechanical strength to that of the natural corneal tissue. When the other conditions were the same, the mechanical strength and light transmittance of the aligned scaffold were much better than the random ones. After seeding the cells, the HKs and HCECs both adhered and proliferated well on the PVA-COL scaffolds; interacted more favorably on the aligned scaffolds; and the aligned nanofibers induced orderly HK growth but did not induce the ordered growth of the HCEC.

## Figures and Tables

**Table 1:** The average diameter of non-aligned and aligned electrospun nanofibers.

Diameter (nm)	7% Collagen	10% PVA	7% PVA-COL	9% PVA-COL
Non-aligned	301.5 ± 74.2	302 ± 37.9	211.6 ± 142.5	262.9 ± 199.3
Aligned	431.8 ± 72.6	204 ± 55	183.3 ± 96.7	163.1 ± 103.2
